# A Blockchain-Based Authentication and Dynamic Group Key Agreement Protocol

**DOI:** 10.3390/s20174835

**Published:** 2020-08-27

**Authors:** Zisang Xu, Feng Li, Han Deng, Minfu Tan, Jixin Zhang, Jianbo Xu

**Affiliations:** 1 Computer and Communication Engineer Institute, Changsha University of Science and Technology, Changsha 410114, China; lif@csust.edu.cn; 2 Big data development and Research Center, Guangzhou College of Technology and Business, Guangzhou 528138, China; aviva_daisy@163.com (H.D.); 13117329002@163.com (M.T.); 3 School of Computer Science, Hubei University of Technology, Wuhan 430068, China; zhangjixin@hnu.edu.cn; 4 School of Computer Science and Engineering, Hunan University of Science and Technology, Xiangtan 411201, China; jbxu@hnust.edu.cn

**Keywords:** authentication, blockchain, cryptography, group key agreement

## Abstract

With the rapid development of mobile networks, there are more and more application scenarios that require group communication. For example, in mobile edge computing, group communication can be used to transmit messages to all group members with minimal resources. The group key directly affects the security of the group communication. Most existing group key agreement protocols are often flawed in performance, scalability, forward or backward secrecy, or single node failure. Therefore, this paper proposes a blockchain-based authentication and dynamic group key agreement protocol. With our protocol, each group member only needs to authenticate its left neighbor once to complete the authentication, which improved authentication efficiency. In addition, our protocol guarantees the forward secrecy of group members after joining the group and the backward secrecy of group members after leaving the group. Based on blockchain technology, we solve the problem of single node failure. Furthermore, we use mathematics to prove the correctness and security of our protocol, and the comparison to related protocols shows that our protocol reduces computation and communication costs.

## 1. Introduction

With the rapid development of mobile networks, the secure transmission of data is no longer limited to both parties in communication, but is required in group communication. Group communication can transmit messages to all group members with minimal resources [1]. This is because the sending of the message only needs to be broadcast once within the group, instead of sending the same message to all group members one by one, which results in a significant increase in communication efficiency. In order to reduce network latency in cloud computing, Mobile Edge Computing (MEC) [2] is introduced. In MEC, the Small Cell Manager (SCM) needs to dynamically and elastically manage the computing and/or storage resources of multiple Small Cell Clouds (SCCs) [3]. Therefore, the use of group communication can greatly improve the efficiency of MEC.

In order to provide a reliable and scalable group communication service, the most basic and critical security issue is access control [4]. In most cases, access control can be achieved by encrypting or decrypting messages, because only legitimate group members can get the key and use this to decrypt the ciphertext to access the messages [5,6]. Therefore, in order to ensure efficient and secure communication in the group, all group members need to use the same session key, which is also called the group key. This means that the group key agreement protocol directly affects the security and efficiency of group communication.

In recent years, many researchers have proposed many authentication and group key agreement protocols. However, according to the research of [7,8], we found that most of these protocols have the following defects: (1) performance: before the group key is negotiated, mutual authentication is usually required between group members, which may consume much of the computation and communication costs; (2) scalability: the protocol cannot efficiently handle the joining or leaving of group members, which results in the poor scalability of the protocol; (3) forward or backward secrecy: it is difficult to guarantee the forward or backward secrecy of group members after joining or leaving the group, such as [9]; (4) single node failure: since most existing protocols store the registration information of all group members in a single node, these protocols are vulnerable to the problem of single node failure. Therefore, these protocols are not suitable for MEC. We design a blockchain-based authentication and dynamic group key agreement protocol to solve the above problems in this paper. The protocol has the following characteristics:

- In our protocol, before negotiating the group key, each group member only needs to authenticate its left neighbor once and perform batch authentication once, instead of implementing mutual authentication between group members, which reduces much of the computation and communication costs.
- The blockchain can be regarded as a shared distributed ledger [10], which can effectively solve the problem of single node failure. Therefore, we use the blockchain to store the public parameters and registration information of all group members. This allows our protocol to solve the problem of single node failure, while also making all the parameters and information stored in the blockchain unmodifiable [11]. In addition, based on blockchain technology, group members can join any group in the entire system after completing registration on any server, which improves convenience.
- In our protocol, when group members join or leave a group, they only needs to update the parameters of an adjacent group member, which also improves the scalability of our protocol.
- Our protocol guarantees the forward secrecy of group members after joining the group and the backward secrecy of group members after leaving the group.

The rest of this article is arranged as follows. Section 2 describes the related works. Some preliminaries are introduced in Section 3. The proposed protocol is described completely in Section 4. Section 5 analyzes the security of our protocol. The performance analysis is shown in Section 6. Finally, we conclude the article in Section 7.

## 2. Related Works

Many researchers have proposed many solutions to ensure the security of group keys. These solutions are generally divided into the following three types [12,13].

(1) Centralized group key agreement protocol:

There is usually only one entity for controlling the entire group in this type of protocol, which is called a Key Distribution Center (KDC). The KDC is responsible for key generation, distribution, and management. It also needs to be responsible for tasks such as group communication. The protocol proposed by Wong et al. [14] is a typical group key agreement protocol based on a Logical Key Hierarchy (LKH). A binary tree is stored in the KDC of this type of protocol. The root node of the tree is a shared encryption key; the intermediate nodes of the tree hold different keys along the path from the leaves to themselves; and the leaf nodes of the tree hold keys related to group members. This type of protocol requires less space to store the keys, and when the keys need to be updated, the amount of communication is greatly reduced. The group key agreement protocol of Islam et al. [7] is proposed for the Internet of Vehicles, and the trusted authority in their protocol acts as the KDC. In the centralized group key agreement protocol, since the KDC needs to be responsible for storing and distributing all keys and controlling group communication, the scalability and storage costs of this type of protocol are very large. In addition, once the KDC fails, the entire system cannot continue to operate normally.

(2) Decentralized group key agreement protocol:

This type of protocol usually divides the entire group into several subgroups, and each subgroup has a group controller to manage all group members in the subgroup. In this way, the burden of KDC is greatly reduced, and single node failure is also solved. Mittra [15] proposed a scalable multicast framework, which divides large groups into multiple subgroups, and each subgroup has a controller called a group security intermediate node or a group security agent. In the protocol of Setia et al. [16], the group key is updated at regular intervals, rather than when group members join or leave. Naresh et al. [8] proposed a cluster-based hybrid group key agreement protocol, which divides large groups into a certain number of clusters and specifies the last member of the cluster as the cluster head and group controller. In 2018, Gupta et al. [17] proposed a group key agreement protocol based on self-certified public keys. In their protocol, there is a group controller for each group. Moreover, before generating the group key, each group member needs to mutually authenticate with other group members, which leads to high computation and communication costs. Zheng et al. [18] proposed a multi-domain group key agreement protocol. Their protocol uses authentication between group members and a group controller instead of authentication between each group member to reduce computation and communication costs. However, this makes their protocol vulnerable to single node failure. The main problems faced by decentralized group key agreement protocols are key distribution efficiency, how to establish a trusted relationship with a third party, and mutual authentication of sub-members across groups.

(3) Distributed group key agreement protocol:

All group members in this type of protocol are equal, and there is no group controller. In addition, the KDC usually does not participate in the generation of group keys. Without a base station, Wang et al. [19] proposed a device-to-device group key agreement protocol. The protocol guarantees the anonymity of each device and uses a signature scheme based on the gap Diffie–Hellman group [20]. In 2018, Zhang et al. [21] proposed a distributed group key agreement protocol. Their agreement achieves cross-domain authentication and key self-certification. Based on the hyper elliptic curve digital signature and ElGamal algorithm, Kavitha et al. [22] proposed a distributed group authentication protocol for the healthcare system in the Internet of Things. Since the KDC or group controller in this type of protocol usually does not participate in the process of group key generation, the mutual authentication between group members becomes the largest computational overhead, which means that the cost of generating a group key will increase as the number of group members increases. Therefore, reducing the number of mutual authentications between group members is the core of reducing the cost of generating a group key. There are many protocols dedicated to reducing the number of authentications between group members by reducing the number of authentication rounds. The group key agreement protocol proposed by Geng et al. [23] and Zheng et al. [9] divides the entire protocol into two rounds. The first round is mutual authentication between members, and the second round is group key generation. In the above two protocols, each group member only needs to authenticate the two adjacent group members. The protocol proposed by Zhang et al. [24] and Shi et al. [25] merges the two processes described above into one. The protocol proposed by Alphonse and Reddy [26] forms each group member into a structure similar to a binary tree, and all group members authenticate each other from the leaf node to the root node. Although their protocol reduces the computation and communication costs required to generate group keys, all group members must wait for the final root node to be authenticated before they can negotiate the group key. Therefore, the computing time is still not low.

## 3. Preliminaries

### 3.1. Network Model

**Figure 1:**
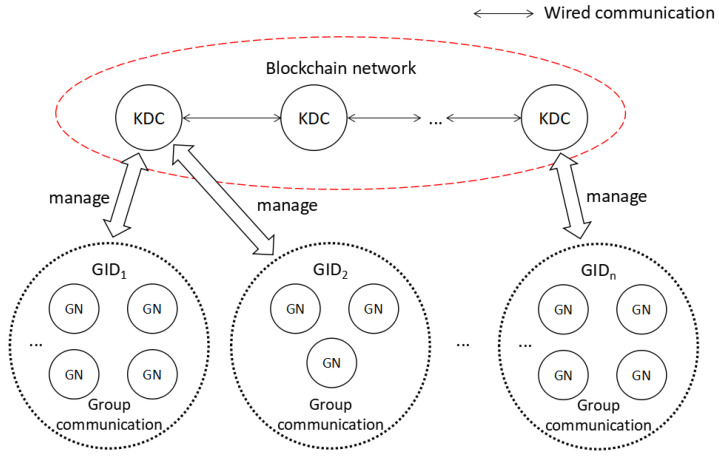
The network model used by our protocol.

There are two parts in our network model, namely KDC and General Node (GN). In MEC, the KDC can be regarded as the SCM, and the GN can be regarded as the SCC. All GNs are equal, and there is no hierarchy or subordinate relationship. In addition, all GNs usually have certain computing and storage resources, and they can join or leave a group at any time. All KDCs are wire connected, and each KDC can manage one or more GNs. The network model used in our protocol is shown in Figure 1.

In our protocol, multiple KDCs form a blockchain network. In order to improve the efficiency of new block generation, we consider using a more efficient Proof-of-Stake (PoS) [27] or Delegated Proof-of-Stake (DPoS) [28] consensus mechanism, such as ouroboros, a provably secure PoS protocol [27], instead of using a Proof-of-Work (PoW) mechanism [29]. According to this consensus mechanism, at regular intervals, all KDCs will regenerate new blocks including groups whose GNs have changed during this period. In each block, in addition to the hash value of the previous block, the timestamp, and Merkle tree root, it also contains the identifier of these groups, the identity list of all GNs in these group, and the related parameters of all GNs in these group. All GNs only have the permission to read information from the blockchain. In addition, there may be multiple different groups, so after the GN enters the network, it first needs to select a group to join.

Before the GN joins the network, it can submit its identity to a KDC closest to it. The KDC will calculate a pair of keys based on the identity and distribute it to the GN. After that, all KDCs will generate a new block containing the identity and related information of the newly added GN through the consensus mechanism. The detailed operation of KDCs is described in the next section. Note that not all KDCs participate in group key agreement.

### 3.2. Threat Model

We define the threat model as follows:

- The adversary has the ability to intercept all data transmitted over unsecured channels, and he/she can inject new data and replace or replay the previously sent data.
- All KDCs are semi-trusted parties, which means that they may misbehave themselves, but do not conspire with any other KDC [30].
- With the help of a Tamper-Proof Device (TPD), we assume that even if the adversary compromises the KDC, he/she cannot extract any secret keys from it.
- The adversary has the ability to capture any number of GNs and can access all the secret information stored in the GN’s memory by capturing attack.

### 3.3. Bilinear Pairing

Let $G_{1}$ and $G_{2}$ be cyclic additive and multiplicative groups of prime order *q*, respectively. The generator of $G_{1}$ is $g_{1}$. Let $e:G_{1}\times G_{1}\to G_{2}$ be a bilinear pairing, which satisfies the following properties:

- Bilinearity: $\forall P,Q\in G_{1}$ and $\forall a,b\in Z_{q}^{*}$, $e\left(aP,bQ\right)=e{\left(P,bQ\right)}^{a}=e{\left(aP,Q\right)}^{b}=e{\left(P,Q\right)}^{ab}$ are satisfied.
- Non-degenerate: $\forall P,Q\in G_{1}$ such that e(P,Q)≠1.
- Computable: for all $P,Q\in G_{1}$, there is always an effective algorithm to compute e(P,Q).

The security of our protocol is based on the following computationally infeasible problems.

- Elliptic Curve Discrete Logarithm problem (ECDL): Let $a\in Z_{q}^{*}$, given $P,aP\in G_{1}$, and compute *a*.
- Computational Diffie–Hellman problem (CDH): Let $a,b\in Z_{q}^{*}$, given $g_{1}$, $ag_{1}$, and $bg_{1}$, and find $abg_{1}$.
- Decisional Diffie–Hellman problem (DDH): Let $a,b,c\in Z_{q}^{*}$, given $g_{1}$, $ag_{1}$, $bg_{1}$, and $cg_{1}$, and decide if $e\left(ag_{1},bg_{1}\right)=e\left(g_{1},cg_{1}\right)$.

## 4. Proposed Protocol

In the distributed group key agreement protocol, each group member is equal, which means that before the group key is negotiated, each group member usually needs to consume many communication and computing resources to perform mutual authentication with all other group members. In order to reduce these costs, in our protocol, we arrange all GNs into a list according to their identities. According to the list, before the group key is negotiated, each GN only needs to send an authentication request to its right neighbor once and be authenticated by its right neighbor. In other words, each GN will receive an authentication request from its left neighbor and authenticate its left neighbor. Since each GN only needs to complete authentication once, this can greatly reduce the computation and communication costs caused by authentication between group members. In addition, when any GN needs to join or leave the group, only the left neighbor of the GN needs to update the parameters, which can also reduce the computation and communication overhead.

Our protocol has seven parts: the initialization phase, the registration phase, the mutual authentication phase, the group key generation phase, the GN join phase, the GN leave phase, and the internal attacker detection process. When the system runs for the first time, the initialization phase is performed by the System Administrator (SA). Each GN performs the registration phase before entering the network. When the group key needs to be negotiated, all GNs perform the mutual authentication phase and the group key generation phase. When a GN wants to join a group, it needs to perform the GN join phase. When a GN in the group wants to leave, the GN leave phase is performed. If the group key fails to be generated multiple times, the KDC will execute the internal attacker detection process to find the malicious GN and expel it from the group.

Suppose there are $GN_{i}\left(1\leq i\leq n\right)$ that need to generate the group key, and their identities are $ID_{i}\left(1\leq i\leq n\right)$, where *n* is the number of GN. Since there may be multiple groups, we named each group $GID_{u}$, where *u* is the number of groups. Each group has a list *L* that stores the identity $ID_{i}$ of all GNs in the group and is managed by the KDC. All $ID_{i}$ in *L* are sorted in descending order, and *L* is a circular list, which means that the largest $ID_{i}$ and the smallest $ID_{i}$ are linked. Table 1 shows the description of the symbols. The details of the above seven parts are as follows.

### 4.1. Initialization Phase

First, the SA picks $\left\{G_{1},G_{2},Q,e,p\right\}$, where $G_{1}$ is a cyclic additive group of order *p*, $G_{2}$ is a cyclic multiplicative group of order *p*, *Q* is a generator of $G_{1}$, and $e:G_{1}\times G_{1}\to G_{2}$ is a bilinear map. Second, the SA generates a random private key *s* and computes the corresponding public key $P_{pub}=sQ$. Finally, the SA publishes parameters {*p*, $G_{1}$, $G_{2}$, *Q*, *e*, $P_{pub}$, h(.), $E_{k}$, $D_{k}$} and stores *s* in the memory of each KDC in a secure environment, where h(.) is the hash function used by this protocol, $E_{k}$ is the symmetric encryption algorithm, and $D_{k}$ is the symmetric decryption algorithm.

### 4.2. Registration Phase

Before the GN joins the network, it needs to choose the nearest KDC to register and receive the corresponding key. The steps in this phase are as follows.

**Step R1:** The KDC generates a unique identity $ID_{i}$ for the $GN_{i}$ and computes its public key $W_{i}=h\left(ID_{i}\right)$ and the corresponding private key $S_{i}=sW_{i}$. Finally, the KDC sends $S_{i}$ to $GN_{i}$ through a secure channel.

**Step R2:**$GN_{i}$ generates a random $a_{i}$, computes $A_{i}=a_{i}Q$, and sends $A_{i}$ to the KDC through a secure channel.

**Step R3:** The KDC broadcasts $ID_{i}$, $W_{i}$, and $A_{i}$.

**Figure 2:**
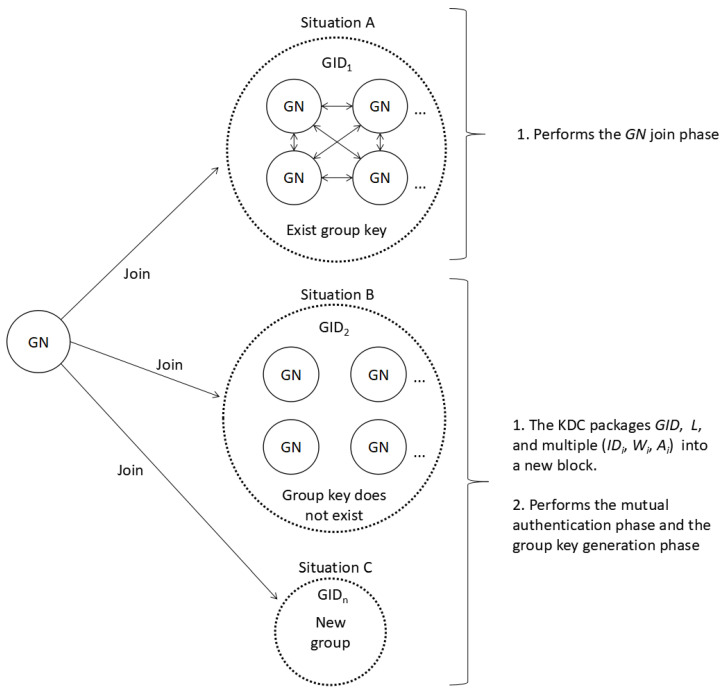
The three situations faced by the GN and the solution strategies.

**Step R4:** According to the group’s identity GID, the GN can choose to join a group or pick a new unique GID to create a new group. Based on the selection of the GN, there are three different situations: (1) Situation A: the GN joins a group that already has a group key; (2) Situation B: the GN joins a group that has not started negotiating group keys; (3) Situation C: the GN creates a new group. In Situation A, the GN performs the GN join phase. In Situation B and Situation C, the KDC will package GID, the corresponding *L*, and multiple tuples ($ID_{i}$, $W_{i}$, $A_{i}$) related to all members of the group into a new block, which will be verified by all other KDCs. After successful verification, the new block will be linked to the blockchain. Figure 2 shows the three situations faced by the GN and the solution strategies.

### 4.3. Mutual Authentication Phase

**Figure 3:**
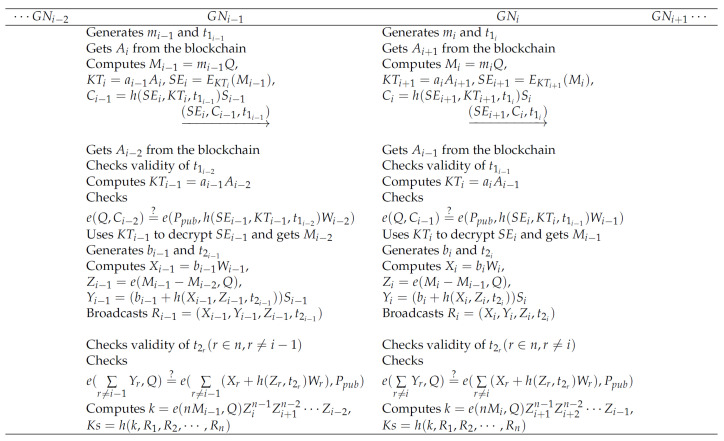
The mutual authentication phase and the group key generation phase of our protocol.

In this phase, $GN_{i}$ first sends a message to its right neighbor $GN_{i+1}$, and $GN_{i+1}$ will authenticate $GN_{i}$. At the same time, $GN_{i}$ will also receive a message from its left neighbor $GN_{i-1}$, and it needs to authenticate $GN_{i-1}$. Figure 3 shows the mutual authentication phase and the group key generation phase of our protocol. $GN_{i}$ performs the following operations.

**Step A1:** Generates a random $m_{i}$ and a timestamp $t_{1_{i}}$ and gets $A_{i+1}$ from the blockchain.

**Step A2:** Computes $M_{i}=m_{i}Q$, $KT_{i+1}=a_{i}A_{i+1}$, $SE_{i+1}=E_{KT_{i+1}}\left(M_{i}\right)$, $C_{i}=h\left(SE_{i+1},KT_{i+1},t_{1_{i}}\right)S_{i}$.

**Step A3:** Sends message ($SE_{i+1}$, $C_{i}$, $t_{1_{i}}$) to $GN_{i+1}$.

**Step A4:**$GN_{i}$ receives a message ($SE_{i}$, $C_{i-1}$, $t_{1_{i-1}}$) from $GN_{i-1}$ and gets $A_{i-1}$ from the blockchain.

**Step A5:** Checks that $t_{new}-t_{1_{i-1}}<\Delta t$ holds or not. If the check fails, it broadcasts an authentication failure message.

**Step A6:** Computes $KT_{i}=a_{i}A_{i-1}$.

**Step A7:** Checks whether the condition $e\left(Q,C_{i-1}\right)?=e\left(P_{pub},h\left(SE_{i},KT_{i},t_{1_{i-1}}\right)W_{i-1}\right)$ is satisfied. If the condition is not true, it broadcasts an authentication failure message.

**Step A8:** Uses $KT_{i}$ to decrypt $SE_{i}$ and get $M_{i-1}$.

**Step A9:** Generates a random $b_{i}$ and a timestamp $t_{2_{i}}$.

**Step A10:** Computes $X_{i}=b_{i}W_{i}$, $Z_{i}=e\left(M_{i}-M_{i-1},Q\right)$, $Y_{i}=\left(b_{i}+h\left(X_{i},Z_{i},t_{2_{i}}\right)\right)S_{i}$.

**Step A11:** Broadcasts $R_{i}=\left(X_{i},Y_{i},Z_{i},t_{2_{i}}\right)$.

### 4.4. Group Key Generation Phase

During this phase, each $GN_{i}$ receives the message $R_{r}\left(r\in n,r\neq i\right)$ from all other GNs. At this point, each $GN_{i}$ will perform a group authentication and then negotiate the group key. The execution steps of each $GN_{i}$ are as follows.

**Step K1:** Checks that the timestamp $t_{new}-t_{2_{r}}<\Delta t,\left(r\in n,r\neq i\right)$ in each received message is valid. If the check fails, it broadcasts an authentication failure message.

**Step K2:** After receiving the message from all other GNs, it checks that:

$$\begin{array}{c} e\left(\sum_{r\neq i}Y_{r},Q\right)\overset{?}{=}e\left(\sum_{r\neq i}\left(X_{r}+h\left(Z_{r},t_{2_{r}}\right)W_{r}\right),P_{pub}\right) \end{array}$$

holds or not. If the check fails, it broadcasts an authentication failure message.

**Step K3:** Computes $k=e\left(nM_{i},Q\right)Z_{i+1}^{n-1}Z_{i+2}^{n-2}\cdots Z_{i-1}$ and group key $Ks=h(k,R_{1},R_{2},\cdots ,R_{n})$.

### 4.5. GN Join Phase

**Figure 4:**
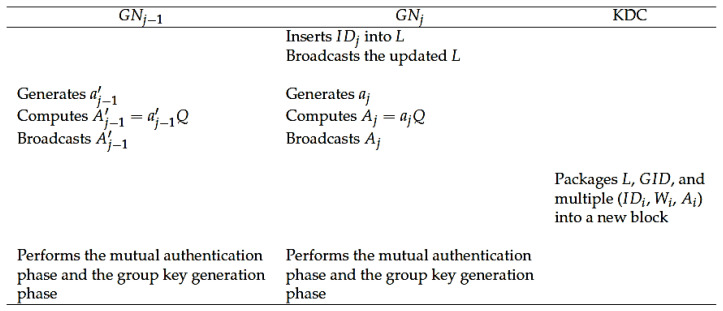
The GN join phase of our protocol.

When a new $GN_{j}$ wants to join the group, it needs to be registered in the KDC first. This means that according to the registration phase, $GN_{j}$ has selected a group to join. Figure 4 shows the GN join phase of our protocol. The detailed steps are as follows.

**Step J1:**$GN_{j}$ obtains the corresponding list *L* of the group from the blockchain, inserts its identity $ID_{j}$ into the appropriate position in *L*, and broadcasts the new *L*.

**Step J2:**$GN_{j}$ generates a random number $a_{j}$ and computes $A_{j}=a_{j}Q$. After that, $GN_{j}$ broadcasts $A_{j}$.

**Step J3:**$GN_{j}$’s left neighbor $GN_{j-1}$ regenerates a new random number $a_{j-1}^{\prime }$ and computes $A_{j-1}^{\prime }=a_{j-1}^{\prime }Q$. After that, $GN_{j-1}$ broadcasts $A_{j-1}^{\prime }$.

**Step J4:** The KDC packages the updated *L*, corresponding GID, and multiple tuples ($ID_{i}$, $W_{i}$, $A_{i}$) related to all members of the group into a new block, which will be verified by all other KDCs. After successful verification, the new block will be linked to the blockchain.

**Step J5:** According to the steps in the mutual authentication phase, $GN_{j}$ sends ($SE_{j+1}$, $C_{j}$, $t_{1_{j}}$) to its right neighbor $GN_{j+1}$ and receives message ($SE_{j}^{\prime }$, $C_{j_{1}}^{\prime }$, $t_{1_{j-1}}^{\prime }$) from $GN_{j-1}$.

**Step J6:** After the messages received by $GN_{j}$ and $GN_{j+1}$ are successfully authenticated, all GNs broadcast $R_{i}=\left(X_{i},Y_{i},Z_{i},t_{2_{i}}\right)$ and perform the group key generation phase to complete the key update.

### 4.6. GN Leave Phase

**Figure 5:**
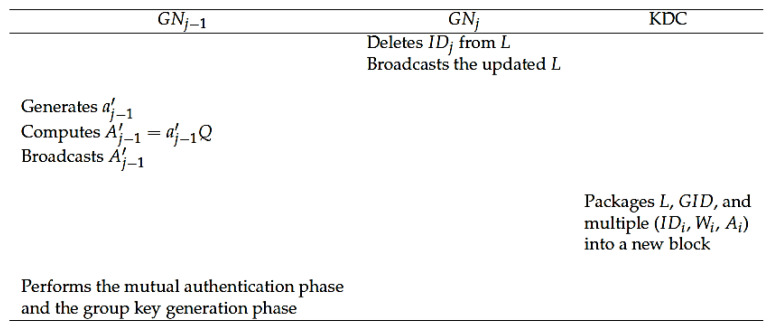
The GN leave phase of our protocol.

When a $GN_{j}$ in the group wants to leave, the following steps need to be performed. Figure 5 shows the GN leave phase of our protocol.

**Step L1:**$GN_{j}$ deletes its identity $ID_{j}$ from list *L* and broadcasts the new *L*.

**Step L2:**$GN_{j-1}$ regenerates a new random number $a_{j-1}^{\prime }$, and computes $A_{j-1}^{\prime }=a_{j-1}^{\prime }Q$. After that, $GN_{j-1}$ broadcasts $A_{j-1}^{\prime }$.

**Step L3:** The KDC packages the updated *L*, corresponding GID, and multiple tuples ($ID_{i}$, $W_{i}$, $A_{i}$) related to all members of the group into a new block, which will be verified by all other KDCs. After successful verification, the new block will be linked to the blockchain.

**Step L4:** According to the steps in the mutual authentication phase, $GN_{j-1}$ sends ($SE_{j}^{\prime }$, $D_{j-1}^{\prime }$, $t_{1_{j-1}}^{\prime }$) to $GN_{j+1}$.

**Step L5:** After $U_{j+1}$ authenticates $U_{j-1}$, all GNs broadcast $R_{i}=\left(X_{i},Y_{i},Z_{i},t_{2_{i}}\right)$ and perform the group key generation phase to complete the key update.

### 4.7. Internal Attacker Detection Process

It can be found in our protocol that every GN is required to be honest during the group key generation phase. If a malicious GN intentionally broadcasts an error message, the entire group cannot generate a group key. However, since all GNs are equal, it is impossible to discover malicious GNs through these GNs. Therefore, our agreement regards KDC as the judging body. When the group fails to negotiate multiple times, the process will be executed and try to find the malicious GN. The steps of this process are as follows.

**Step D1:** The KDC first records all messages sent by each $GN_{i}$ in the group GID.

**Step D2:** All $GN_{i}$ generate a timestamp $t_{D_{i}}$, compute $H=h(W_{i},S_{i},ID_{i},KT_{i+1},b_{i})$, and send the tuple ($ID_{i}$, $W_{i}$, *H*, $KT_{i+1}$, $b_{i}$) to the KDC.

**Step D3:** After receiving the tuple, the KDC first verifies the validity of the time stamp $t_{D_{i}}$. Then, the KDC computes $S_{i}^{\prime }=sW_{i}$, $H^{\prime }=h\left(W_{i},S_{i}^{\prime },ID_{i},KT_{i+1},b_{i}\right)$ and verifies $H^{\prime }?=H$. If it does not hold, $GN_{i}$ is regarded as a malicious GN.

**Step D4:** If the above formula holds, the KDC continues to compute $C_{i}^{\prime }=h\left(SE_{i+1},KT_{i+1},t_{1_{i}}\right)S_{i}$, $Y_{i}^{\prime }=\left(b_{i}+h\left(X_{i},Z_{i},t_{2_{i}}\right)\right)S_{i}$ and verify $C_{i}^{\prime }?=C_{i}$, $Y_{i}^{\prime }?=Y_{i}$. If it does not hold, the corresponding $GN_{i}$ will be expelled from the group immediately.

## 5. Security and Performance Analysis

### 5.1. Correctness Analysis

**Theorem** **1.** *$GN_{i}$ and $GN_{i+1}$ can calculate the same symmetric key $KT_{i+1}$, so that $GN_{i+1}$ can get $M_{i}$.*

**Proof.**  Since $GN_{i}$ computes $KT_{i+1}=a_{i}A_{i+1}$ to get $KT_{i+1}$ and $GN_{i+1}$ computes $KT_{i+1}=a_{i+1}A_{i}$ to get $KT_{i+1}$, then:

$$\begin{array}{c} \begin{array}{cc} KT_{i+1} & =a_{i}A_{i+1}\\ & =a_{i}a_{i+1}Q\\ & =a_{i+1}A_{i}. \end{array} \end{array}$$

Since the same $KT_{i+1}$ can be obtained by calculating $a_{i}A_{i+1}$ and $a_{i+1}A_{i}$, $GN_{i}$ and $GN_{i+1}$ can use the symmetric key $KT_{i+1}$ to encrypt or decrypt transmitted messages. □

**Theorem** **2.** *It is valid for $GN_{i}$ to authenticate $GN_{i-1}$.*

**Proof.**  The authentication of $GN_{i}$ for $GN_{i-1}$ is achieved by verifying whether the formula $e\left(Q,C_{i-1}\right)=e\left(P_{pub},h\left(SE_{i},KT_{i},t_{1_{i-1}}\right)W_{i-1}\right)$ holds. The correctness of the formula is proven as follows.

$$\begin{array}{c} \begin{array}{cc} e(Q,C_{i-1}) & =e(Q,h\left(SE_{i},KT_{i},t_{1_{i-1}}\right)S_{i-1})\\ & =e(Q,h\left(SE_{i},KT_{i},t_{1_{i-1}}\right)sW_{i-1})\\ & =e(P_{pub},h\left(SE_{i},KT_{i},t_{1_{i-1}}\right)W_{i-1}) \end{array} \end{array}$$

Although the adversary can easily obtain $Q,P_{pub},A_{i}\in G_{1}$, due to the ECDL and the CDH, he/she cannot calculate *s*, any $a_{i}$, or $KT_{i}$ in polynomial time. Therefore, the adversary also cannot calculate $h(SE_{i},KT_{i},t_{1_{i-1}})$. If the adversary wants to forge a $GN_{i}$ to pass the above verification, he/she needs to create a new valid $e(P_{pub},h\left(SE_{i},KT_{i},t_{1_{i-1}}\right)W_{i-1})$. However, since the adversary cannot obtain $a_{i}$ and *s*, he/she cannot calculate a new $e(P_{pub},h\left(SE_{i},KT_{i},t_{1_{i-1}}\right)W_{i-1})$. In addition, due to the DDH, for a random $z\in G_{1}$, the adversary cannot decide if $e\left(Q,C_{i-1}\right)=e\left(P_{pub},z\right)$ in polynomial time. □

**Theorem** **3.** *During the group key generation phase, $GN_{i}$ is valid for the batch authentication of other group members.*

**Proof.**  In the group key generation phase, $GN_{i}$ authenticates other group members in batches by verifying whether formula $e\left(\sum_{r\neq i}Y_{r},Q\right)=e\left(\sum_{r\neq i}\left(X_{r}+h\left(X_{r},Z_{r},t_{2_{r}}\right)W_{r}\right),P_{pub}\right)$ holds. The correctness of the formula is proven as follows.

$$\begin{array}{c} \begin{array}{cc} e(\sum_{r\neq i}Y_{r},Q) & =e(\sum_{r\neq i}\left(b_{r}+h\left(X_{r},Z_{r},t_{2_{r}}\right)\right)S_{r},Q)\\ & =e(\sum_{r\neq i}\left(b_{r}+h\left(X_{r},Z_{r},t_{2_{r}}\right)\right)sW_{r},Q)\\ & =e(\sum_{r\neq i}\left(b_{r}W_{r}+h\left(X_{r},Z_{r},t_{2_{r}}\right)W_{r}\right),sQ)\\ & =e(\sum_{r\neq i}\left(X_{r}+h\left(X_{r},Z_{r},t_{2_{r}}\right)W_{r}\right),P_{pub}). \end{array} \end{array}$$

If the adversary wants to forge a $GN_{i}^{*}$ to pass the above batch authentication, he/she needs to create a valid $X_{i}^{*}$ and $Y_{i}^{*}$ to satisfy $e\left(Y_{i}^{*},Q\right)=e\left(\left(b_{i}+h\left(X_{i}^{*},Z_{i},t_{2_{i}}\right)\right)sW_{i},Q\right)$. First, the adversary cannot get $b_{i}$, so it is difficult for him/her to calculate $\left(b_{i}+h\left(X_{i}^{*},Z_{i},t_{2_{i}}\right)\right)sW_{i}$. Second, even suppose that $\left(b_{i}+h\left(X_{i}^{*},Z_{i},t_{2_{i}}\right)\right)$ is revealed by the adversary, but he/she still cannot calculate a valid $X_{i}^{*}$ or $Y_{i}^{*}$ because he/she cannot get *s*. In addition, due to the DDH, for a random $z\in G_{1}$, the adversary cannot decide if $e\left(\sum_{r\neq i}Y_{r},Q\right)=e\left(z,P_{pub}\right)$ in polynomial time. □

**Theorem** **4.** *If all $GN_{i}$s participating in the group key generation phase are honest, then all $GN_{i}$s can negotiate the same group key.*

**Proof.**  According to Theorem 1, as long as all $GN_{i}$s participating in the group key generation phase are honest, each $GN_{i}$ can obtain the parameter $M_{i-1}$ sent by its left neighbor. Therefore,

$$\begin{array}{c} \begin{array}{cc} k & =e\left(nM_{i},Q\right)Z_{i+1}^{n-1}Z_{i+2}^{n-2}\cdots Z_{i-1}\\ & =e{\left(m_{i}Q,Q\right)}^{n}Z_{i+1}^{n-1}Z_{i+2}^{n-2}\cdots Z_{i-1}\\ & =e{\left(Q,Q\right)}^{nm_{i}+\left(n-1\right)\left(m_{i+1}-m_{i}\right)+\left(n-2\right)\left(m_{i+2}-m_{i+1}\right)+\cdots +\left(m_{i-1}-m_{i-2}\right)}\\ & =e{\left(Q,Q\right)}^{m_{1}+m_{2}+\cdots +m_{i}}. \end{array} \end{array}$$

From the above, it can be found that all $GN_{i}$s can calculate the same parameter *k*. Therefore, their group keys $Ks=h(k,R_{1},R_{2},\cdots ,R_{n})$ are also the same. □

### 5.2. Simulation Based on the ProVerif Tool

**Figure 6:**
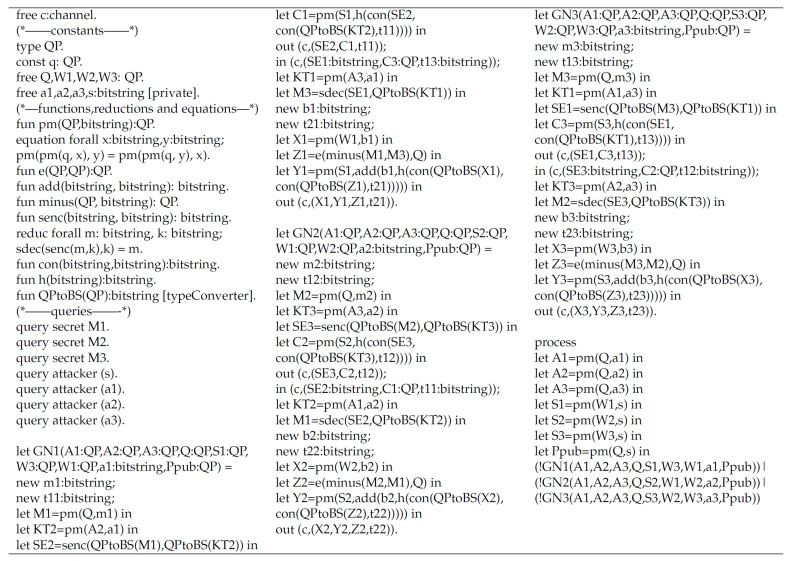
The code for the mutual authentication phase of our protocol.

**Figure 7:**
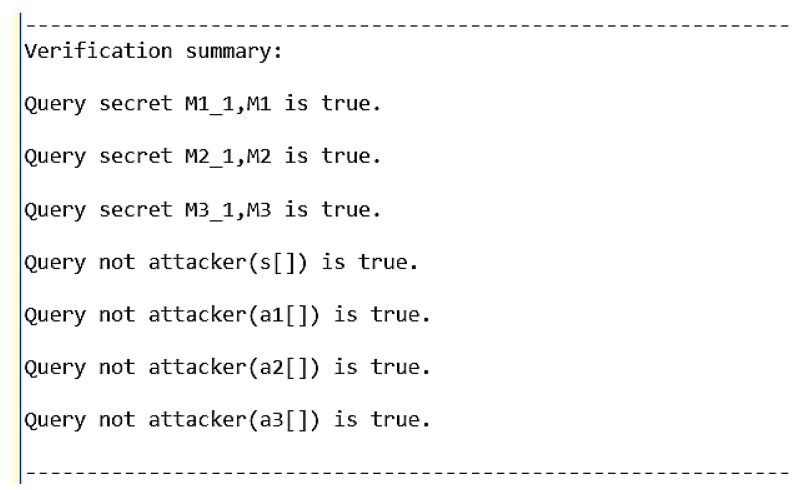
The PV simulation results of our protocol.

ProVerif is a widely known authentication protocol verification tool that can prove the security of multiple encryption schemes or authentication protocols, such as signature schemes and Diffie–Hellman key exchange algorithms [31,32]. Since each GN does not need to communicate with other nodes during the group key generation phase, we use ProVerif to verify the security of the mutual authentication phase of our protocol. In addition, since we do not need to verify the performance of our protocol in this section, we assume that there are only three GNs that need to negotiate a group key, which are $GN_{1}$, $GN_{2}$, and $GN_{3}$. Figure 6 shows the code for the mutual authentication phase of our protocol. Figure 7 shows the simulation results. The results show that in our protocol, the secret parameters $M_{1}$, $M_{2}$, and $M_{3}$ for group key generation, as well as the private keys *s*, $a_{1}$, $a_{2}$, and $a_{3}$ will not be obtained by the adversary.

### 5.3. Informal Security Analysis

#### 5.3.1. GN Impersonation Attack

The adversary needs to create valid messages ($SE_{i+1}$, $C_{i}$, $t_{1_{i}}$) or ($X_{i},Y_{i},Z_{i},t_{2_{i}}$) to perform an impersonation attack on $GN_{i}$. However, according to Theorem 2 and Theorem 3, the adversary cannot create a valid message.

#### 5.3.2. GN Capture Attack

After the adversary has captured several GNs, we need to ensure that the private key *s* cannot be obtained by the adversary. After the adversary captures a $GN_{i}$, he/she can obtain $S_{i}$ containing information about *s*. However, according to the ECDL, although the adversary can obtain $W_{i}$ and $sW_{i}$, he/she cannot calculate *s* in polynomial time.

#### 5.3.3. Replay Attack

Our protocol uses timestamps $t_{1}$ and $t_{2}$ to defend against replay attacks. In addition, our protocol guarantees that the timestamp cannot be modified. For messages ($SE_{i+1}$, $C_{i}$, $t_{1_{i}}$), the adversary must obtain $KT_{i+1}$ and $S_{i}$ before replacing a new valid timestamp $t_{1_{i}}^{\prime }$. For messages ($X_{i},Y_{i},Z_{i},t_{2_{i}}$), the adversary must obtain $b_{i}$ and $S_{i}$ before replacing a new valid timestamp $t_{2_{i}}^{\prime }$. However, the adversary cannot get $KT_{i+1}$, $b_{i}$ or $S_{i}$ because they are never transmitted directly in the channel. In addition, due to the ECDL, it is difficult for the adversary to calculate these parameters.

#### 5.3.4. Forward Secrecy after a New GN Joins

When a GN wants to join a group, it needs to ensure that $GN_{j}$ cannot get the previous group key. In our protocol, we assume that $GN_{j}$ has intercepted all historical communication messages of the group. This results in that when $GN_{j}$ joins the group, as long as it obtains $m_{j-1}$, it can calculate the previous group key. However, when the $GN_{j}$ joins the group, its left neighbor $GN_{j-1}$ will regenerate a new $m_{j-1}^{\prime }$ and send it to $GN_{j}$, which means $GN_{j}$ can only get $m_{j-1}^{\prime }$ instead of $m_{j-1}$. Therefore, our protocol ensures the forward secrecy after a new GN joins.

#### 5.3.5. Backward Secrecy after a GN Leaves

When a $GN_{j}$ leaves the group, it needs to ensure that it cannot get the group key generated in the future. We assume that after $GN_{j}$ leaves the group, it still retains the secret parameter $m_{j-1}$ sent by its left neighbor and is able to intercept all group communication messages. This means that as long as $m_{j-1}$ is kept unchanged, $GN_{j}$ can still calculate the group key after leaving the group. However, in our protocol, after $GN_{j}$ leaves the group, its left neighbor $U_{j-1}$ will regenerate a new $m_{j-1}^{\prime }$ and send it to $GN_{j+1}$. This results in that at Step A11, $GN_{j-1}$ will broadcast $Z_{j-1}^{\prime }=e\left(M_{j-1}^{\prime }-M_{j-2},Q\right)$ and $U_{j+1}$ will broadcast $Z_{j+1}^{\prime }=e\left(M_{j+1}-M_{j-1}^{\prime },Q\right)$. At this point, $m_{j-1}$ has expired, and $GN_{j}$ cannot calculate the group key through this parameter. Therefore, our protocol ensures the backward secrecy after a GN leaves.

#### 5.3.6. Single Node Failure

Although all KDCs will not participate in the mutual authentication phase and the group key generation phase, each group still needs to be managed by a KDC. This is because each group may need the KDC to act as a judging body to expel malicious nodes. If the KDC fails, since all KDCs share the same blockchain that stores all GNs’ authentication parameters, it only needs to switch to another KDC. This means that in our protocol, as long as there is a working KDC, the entire system can operate normally.

## 6. Performance Analysis and Comparison

### 6.1. Computation Cost

The symbols $t_{sym}$, $t_{h}$, $t_{pm}$, $t_{pa}$, and $t_{bp}$ represent the computing time required to implement one symmetric encryption or decryption, one general hash function operation, one point multiplication operation on Elliptic Curve Cryptography (ECC), one point addition operation on ECC, and one bilinear pairing operation, respectively. In the mutual authentication phase and the group key generation phase of our protocol, each GN needs to perform n+7 point multiplication operations on ECC, 4 bilinear pairing operations, n+3 hash operations, 2 symmetric encryption or decryption operations, and n+1 point addition operations on ECC, where *n* is the number of GN. In other words, the total computation cost of each GN is $\left(n+7\right)t_{pm}+4t_{bp}+\left(n+3\right)t_{h}+2t_{sym}+\left(n+1\right)t_{pa}$.

### 6.2. Communication Cost

We assume *C* is 256 bits and timestamp *T* is 64 bits. In the mutual authentication phase of our protocol, each GN needs to send message ($SE_{i+1}$, $C_{i}$, $t_{1_{i}}$) to $GN_{i+1}$ and broadcast ($X_{i}$, $Y_{i}$, $Z_{i}$, $t_{2_{i}}$). Therefore, each GN needs to send messages of length 5C+2T and receive messages of length (3n−1)C+nT. The total communication cost in the the mutual authentication phase and the group key generation phase of our protocol is $\left(3n^{2}+4n\right)C+\left(n^{2}+2n\right)T$.

### 6.3. Comparison with Related Protocols

We compare our protocol with the protocol of Zheng et al. [9], the protocol of Zhang et al. [21], and the protocol of Gupta et al. [17] in terms of computation costs, energy consumption, communication costs, and security.

In the protocol of Zheng et al. [9], the total computation cost required to generate the group key is $\left(n+4\right)t_{pm}+6t_{bp}+\left(n+4\right)t_{h}+\left(3n+1\right)t_{pa}$, and each group member needs to send messages of length 7C+2T and receive messages of length (7C+2T)(n−1). In the protocol of Zhang et al. [21], the total computation cost required to generate the group key is $\left(3n+2\right)t_{pm}+2nt_{bp}$, and each group member needs to send messages of length 4C and receive messages of length 4(n−1)C. In the protocol of Gupta et al. [17], the total computation cost required to generate the group key is $4nt_{pm}+5t_{h}+\left(2n+1\right)t_{pa}$. In their protocol, there is a group controller in each group, which undertakes most of the communication work and causes the communication cost of each group member to be very low. Therefore, we consider that the total length of the messages that need to be sent and received is 6nC+1, rather than the communication cost of each group member. Table 2 shows the comparison of computation cost and communication cost between our protocol and related protocols.

**Figure 8:**
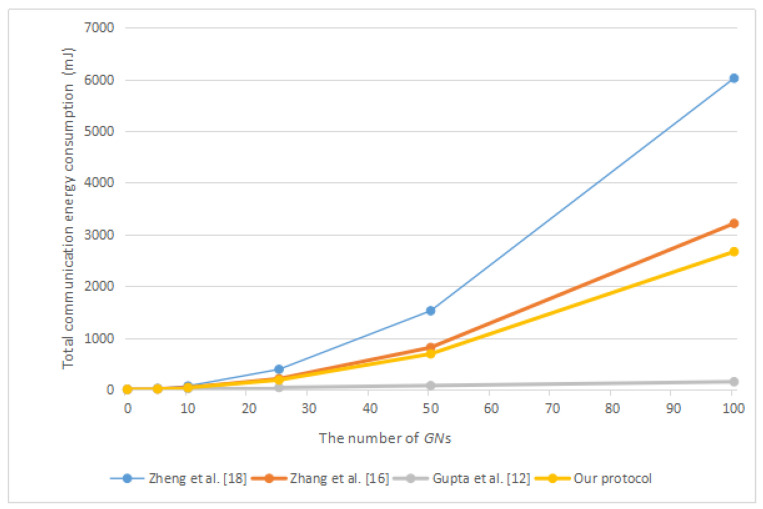
The total communication energy consumption in different protocols.

**Figure 9:**
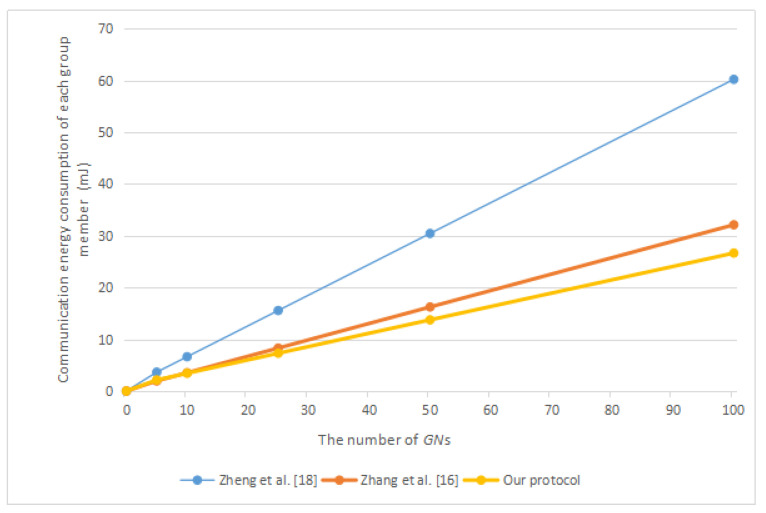
The communication energy consumption of each group member in different protocols.

**Figure 10:**
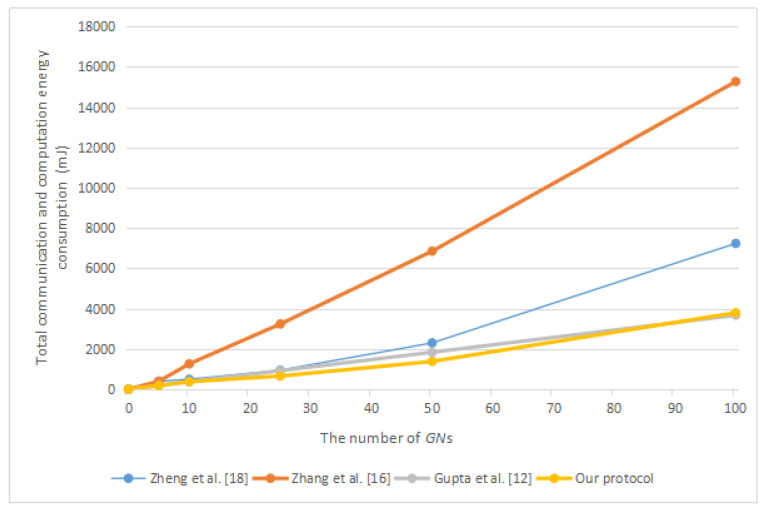
The total communication and computation energy consumption in different protocols.

Next, we will compare our protocol with related protocols in terms of energy consumption. According to the work of Carman et al. [33] and Zhang et al. [21], we obtained that a “Strong ARM” microprocessor running at 133MHz performing one symmetric encryption or decryption operation needs to consume 0.00217 mJ, one point multiplication operation on ECC requires 8.8 mJ, one general hash function operation requires 0.000108 mJ, and one bilinear pairing operation requires 47 mJ. According to the work of Makri and Konstantinou [34], we obtained that an IEEE 802.11 Spectrum24 WLAN card requires 0.00066 mJ for the transmission of 1 bit and 0.00031 mJ for the reception of 1 bit. According to [35], the computing time of one point addition operation on ECC is about half of one symmetric encryption or decryption operation. Therefore, we considered that the energy consumption of a point addition operation is about 0.001085 mJ. We summarize the above energy consumption in Table 3. Figure 8 shows the total communication energy consumption in different protocols. Figure 9 shows the communication energy consumption of each group member in different protocols. Figure 10 shows the total communication and computation energy consumption in different protocols.

It can be found from Table 2 that our protocol has the lowest computation costs, and the computation costs of our protocol and the protocol of Zheng et al. [9] are very similar. However, from Figure 8, it can be found that our protocol consumes less communication resources. In addition, because after group members join or leave a group, neither their left neighbor nor their right neighbor updated the corresponding temporary secret parameter, this leads to the newly joined group members being able to easily obtain the previous group key, and group members who leave the group can also easily obtain the subsequent group key. Therefore, the protocol of Zheng et al. [9] lacks forward or backward secrecy.

According to Figure 10, in the distributed group key agreement protocol, such as the protocol of Zheng et al. [9] and the protocol of Zhang et al. [21], our protocol has the least total communication and computation energy consumption. With the help of the group controller, the decentralized group key agreement protocol such as the protocol of Gupta et al. [17] will consume less energy when there is a large number of group members. However, in the protocol of Gupta et al. [17], each group member needs to perform mutual authentication with the group controller one by one, which requires much total computing time. Moreover, the existence of the group controller makes their protocol vulnerable to single node failure. Once the group controller fails, the group cannot continue to negotiate the group key. According to Figure 8 and Figure 9, excluding the protocol of Gupta et al. [17], the total communication energy consumption of our protocol is the lowest, and the communication energy consumption of each group member in our protocol is also the lowest.

## 7. Conclusions

This paper proposes a blockchain-based authentication and dynamic group key agreement protocol. Each group member in our protocol only needs to authenticate its left neighbor once to complete the authentication, which improves authentication efficiency. When a node joins or leaves a group, only the left neighbor of the node needs to update the data, which also improves the scalability of our protocol. Our protocol also guarantees forward or backward secrecy when group members join or leave the group. In addition, we use blockchain technology to store group identities, a list of group members, and some group member-related parameters, which can solve the problem of single node failure. Finally, we use mathematics and ProVerif to prove the correctness and security of our protocol. The comparison with related protocols shows that our protocol reduces computation and communication costs.

## Figures and Tables

**Table 1:** Symbols used in our protocol.

Symbol	Description
SA	System Administrator
KDC	Key Distribution Center
GN	General node
*q*	A large prime number
$G_{1}$	Cyclic additive groups of prime order *q*
$G_{2}$	Cyclic multiplicative groups of prime order *q*
*Q*	The generator of $G_{1}$
*e*	Bilinear pairing $e:G_{1}\times G_{1}\to G_{2}$
$ID_{i}$	The identity of $GN_{i}$
$GID_{u}$	The identity of the group
*L*	A circular list that stores all GN-related information in the group
*s*	The KDC’s private key
$P_{pub}$	The KDC’s public key
$W_{i}$, $A_{i}$	The GN’s public key
$S_{i}$, $a_{i}$	The GN’s private key
$t_{1}$, $t_{2}$	Timestamp
$t_{new}$	The timestamp when the latest information was received
Δt	Maximum communication transmission delay
$KT_{i}$	Symmetric key
$E_{k}$	Symmetric encryption algorithm
$D_{k}$	Symmetric decryption algorithm
h(.)	Hash operation
Ks	Group key
⊕	Bitwise XOR operation
(a,b)	Concatenation of data *a* and data *b*

**Table 2:** The comparison of computation cost and communication cost between our protocol and related protocols.

	Zheng et al. [9]	Zhang et al. [21]	Gupta et al. [17]	Our Protocol
Point multiplication operations on ECC	n+6	3n+2	4n	n+7
Bilinear pairing	6	2n	0	4
Hash operation	n+9	0	5	n+3
Symmetric encryption or decryption	4	0	0	2
Point addition operations on ECC	3n+1	0	2n+1	3n+1
Message length sent by each group member	7C+2T	4C	-	5C+2T
Message length received by each group member	(7C+2T)(n−1)	4(n−1)C	-	(3n−1)C+nT
Total sent message length	(7C+2T)n	4nC	6nC+1	(5C+2T)n
Total received message length	n(n−1)(7C+2T)	4n(n−1)C	6nC+1	$\left(3n^{2}-n\right)C+n^{2}T$

**Table 3:** Energy consumption for computing and communication.

Operations	Energy Consumption
$t_{sym}$	0.00217 mJ
$t_{pm}$	8.8 mJ
$t_{pa}$	0.001085 mJ
$t_{h}$	0.000108 mJ
$t_{bp}$	47 mJ
Transmitting a bit	0.00066 mJ
Receiving a bit	0.00031 mJ

## Abbreviations

The following abbreviations are used in this manuscript:

MEC	Mobile Edge Computing
SCM	Small Cell Manager
SCC	Small Cell Cloud
GN	General Node
KDC	Key Distribution Center
LKH	Logical Key Hierarchy
TPD	Tamper-Proof Device
ECC	Elliptic Curve Cryptography
SA	System Administrator
ECDL	Elliptic Curve Discrete Logarithm problem
CDH	Computational Diffie–Hellman problem
DDH	Decisional Diffie–Hellman problem
